# Rainfastness of Insecticides Used to Control Spotted-Wing Drosophila in Tart Cherry Production

**DOI:** 10.3390/insects10070203

**Published:** 2019-07-11

**Authors:** Ignatius P. Andika, Christine Vandervoort, John C. Wise

**Affiliations:** 1 Department of Entomology, Michigan State University, East Lansing, MI 48824, USA; 2 Pesticide Analytical Laboratory, Michigan State University, 206 Center for Integrated Plant Systems, Michigan State University, East Lansing, MI 48824-1311, USA

**Keywords:** octanol-water partition coefficient, residue penetration, simulated rainfall

## Abstract

Tart cherry production is challenged by precipitation events that may reduce crop protection against spotted-wing drosophila (*Drosophila suzukii*) (SWD). Due to SWD’s devastating impacts on yield, growers are often faced with the option of insecticide reapplication. Semi-field bioassays were used to assess simulated rainfall effects towards adult mortality, immature survival, and residue wash-off from different plant tissues for several compounds. Tart cherry shoots were treated with 0, 12.7 or 25.4 mm of simulated rainfall and infested with SWD for 5 days. Adult mortality was recorded 1, 3, and 5 days after shoots were infested, while immature stage individuals were counted 9 days after the first infestation day. All insecticides demonstrated higher adult mortality and lower immature survival compared with the untreated control at 0 mm of rainfall. Adult mortality and immature survival caused by phosmet, zeta-cypermethrin, and spinetoram were adversely affected by simulated rainfall. In all bioassays, acetamiprid was the least affected by simulated rainfall. Residue analysis demonstrated phosmet and spinetoram residues to be the most sensitive to wash-off. This study demonstrates different rainfall effects on SWD control for several compounds. This information may provide a basis for making an informed decision on whether reapplication is required.

## 1. Introduction

Spotted-wing drosophila (*Drosophila suzukii* Matsumura) (SWD), a multivoltine polyphagous invasive species originating from East Asia, has become a major fruit pest globally [1]. In 2008, SWD invaded Europe and the United States mainland starting with Spain and California. Since then, reports have stated that SWD has been found in South America, including Uruguay, Brazil, Chile, and Argentina [2,3]. Unlike most other *drosophilids*, female SWD are able to oviposit into the ripening fruit stages of various cultivated soft-skinned fruits, wild and berry fruits, or even decayed durable fruit such as apples or pears using their sclerotized ovipositor, causing earlier infestation during the season [4,5,6]. Therefore, intensive and earlier insecticide spraying programs are required to maintain high-value crops to fulfill market standards [7].

Integrated pest management programs recommend spraying programs start once the target pest is caught in traps at or above a certain threshold level. However, existing traps are impractical and not sufficiently selective to SWD, and also lose competitive attraction as adjacent fruit ripens [8]. This can result in fruit being infested before flies are detected in monitoring traps. Therefore, growers often begin weekly sprays as soon as fruit are susceptible. Currently, US growers are spraying weekly and rotating between organophosphates, spinosyns, and pyrethroids to control SWD [9,10]. These intensive spraying programs may lead to detrimental effects on natural enemies, the environment, and the possibility of developing insecticide resistance within SWD populations. In addition, while growers must comply with seasonal application limitations and global market maximum residue limits, they also have to respond to weather conditions that may interfere with management programs [11,12].

For effective pest control, insecticides must be persistent on or in plant tissues and be able to withstand weather events, such as rainfall, UV light, and temperature [13,14]. Rainfall can have detrimental effects on insecticide performance by dislodging insecticide deposits from the plant surface, decreasing insecticide concentration within plant tissues, and reducing overall insecticide bioavailability to non-lethal doses [15]. Studies have shown that adult SWD mortality decreased with increased rainfall amounts for various insecticides applied on blueberries [16], while others have reported rainfall amount to affect insecticide residue levels [17,18,19]. These studies suggest the impact of rainfall on insecticide performance is influenced by the amount of rain, the inherent toxicity of the compound against the target pest, drying time post-application, affinity of the compound to the plant surface and penetrative capacity, and the physiological attributes of the crop plant. In addition to compromised protection levels, pesticide wash-off could adversely affect the environment [20].

Michigan is the largest tart cherry producing state in the USA, and its leading production counties had an average precipitation amount of 84.75 mm during the growing season over the past 10 years [21]. Growers are faced with a decision of whether or not to reapply insecticides following a precipitation event. Unnecessary reapplication can cause increased production costs and risk of detrimental effects to the environment. Reapplication may also lead to residues exceeding maximum residue limit (MRL) values if fruit are being exported, while not spraying may result in an unprotected crop and SWD infestation at harvest. There are currently no published reports on the impact of rainfall on pesticides used to control pests of tart cherries. The objectives of this study were to investigate the impact of various amounts of rainfall on the performance of insecticides in controlling SWD in tart cherries, both in terms of reducing adult mortality and survival of immature stages and surface and sub-surface insecticide residues from cherry leaves and fruit.

## 2. Materials and Methods

### 2.1. Field Plots and Insecticide Application

Field plots were located at the Michigan State University (MSU) Trevor Nichols Research Center (TNRC) in Fennville, MI, USA (42°35’40.9” N, 86°09’19.9” W). Each treatment plot consisted of one tart cherry tree, *Prunus avium*, cv. Montmorency, surrounded by eight buffer trees (6 m × 4.5 m spacing), replicated five times in a complete randomized design. Insecticide applications were made on 2 July 2018 between 09:00–12:00 with an average air temperature of 21 °C, 88% relative humidity, and 1.29 km/h wind speed. The selected insecticides represented six chemical classes and treatment concentrations were based on labeled field rates (Table 1). Insecticides were applied using an FMC 1029 air-blast sprayer (Jonesboro, AK, USA) calibrated to deliver material and water diluent in 935 L ha^−1^ (100 gallons per acre) of diluent.

### 2.2. Semi-Field Bioassays

Cherry shoots containing 5 fruits and 5 leaves were collected 4 h after treatment and stored in a 2.7 °C walk-in cooler. Cherry shoots were placed in water-soaked OASIS floral foam bricks (Smithers–Oasis Co., Kent, OH, USA) and randomly sorted into a Generation 3 Research Sprayer Track (DeVries Manufacturing, Hollandale, MN, USA). The rainfall simulator was set up with an AI 11008VS nozzle (TeeJet Technologies, Wheaton, IL, USA), run at 69 kPa (10 PSI) and 0.8 Km/h, and the distance between the nozzle and shelf was 100.3 cm. Shoots were run through 12.7 mm or 25.4 mm of simulated rain. Controls (0 mm of simulated rain) were not placed in the rain simulator. Three rain gauges were placed inside the rainfall simulator to measure uniformity and the amount of simulated rainfall. Air-dried cherry shoots were then placed into 0.95 L plastic containers with floral foam on the bottom and food quality wax to ensure shoot stability. Six female and six male spotted-wing drosophila adults were added into containers with a 1 mL diet disc to maintain healthy fly conditions. Adult fly mortality was recorded 1, 3, and 5 days after fly exposure. After 5 days, flies were removed and cherry shoots were held for an additional 4 days until assessment for survival of small larvae (<2 mm), large larvae (>2 mm), and pupae. Fruits were placed into 0.95 L closable bags (Gordon Food Service, Grand Rapids, MI, USA). Fruits were crushed to allow brown sugar water to enter; 150 mL of brown sugar water with a ratio of 172 g of brown sugar per 1 L of tap water was added to each plastic bag. After an hour, the fruit mixtures were poured over a mesh tray with hole spacing of 8.38 mm (SE GP2-14 stackable sifting pan) for larvae and water to run through and cleaned using a washer bottle. Liquid was stored in a 0.95 L plastic container and placed in a walk-in cooler to be assessed the next day, when liquid was poured into a reusable coffee filter and small larvae, large larvae, and pupae were counted under a stereomicroscope. Lethality was determined by comparing total numbers of small larvae, large larvae, pupae, and total individuals found between each insecticide treatment. The bioassay in 2018 was conducted twice to obtain sufficient replications.

### 2.3. Insecticide Residue Analysis

A similar set of shoots to those used in the bioassay were run in the rainfall simulator for residue analysis. Surface and subsurface residues for both leaves and fruit tissue were measured to determine the degree of wash-off due to the simulated rain. Twenty leaves and 10 fruits from each set of treatments were placed, respectively, in 120 mL and 60 mL of acetonitrile high-performance liquid chromatographers (HPLC)-grade solvent (EMD Miliprole Chemicals, Inc., Billerica, MA, USA) and samples were sonicated for 30 s to obtain surface residues. Plant tissues were moved into new sample jars and 120 mL and 60 mL of dichloromethane (VWR Analytical, Radnor, PA, USA) were added, respectively, to leaf and fruit samples. Fruit samples were ground to increase contact surface with solvent. Subsequently, 4 g magnesium sulfate and 1 g sodium chloride were added to leaf and fruit samples. All samples were stored in a 4 °C cooler until laboratory processing at the MSU Pesticide Analytical Laboratory.

#### 2.3.1. Surface Residues

Samples were sonicated for 1 min and acetonitrile was decanted through 12 g sodium sulfate (EMD Chemicals, Inc., Port Wentworth, GA, USA) placed in Whatman filter paper with a diameter of 11.25 cm to remove water (Tisch Scientific, North Blend, OH, USA). Samples were decanted through sodium sulfate again until all water was completely removed. Sodium sulfate columns were rinsed twice with 10 mL of clean acetonitrile to collect remaining residues. Solvent was evaporated under a fume hood and 2 mL of acetonitrile for HPLC or gas chromatography (GC) analysis was added. Samples were sonicated for 1 min to collect any suspected remaining residues. Remaining particulates were removed by passing samples through a 0.45 µm 13 mm syringe filter (Pall, East Hills, NY, USA).

#### 2.3.2. Subsurface Residues

Sample extracts were passed through a column containing 12 g sodium sulfate (EMD Chemicals, Inc., Port Wentworth, GA, USA) placed in Whatman filter paper with a diameter of 11.25 cm to remove remaining water (Tisch Scientific). Filtering using a clean column was repeated until remaining water was collected. Sodium sulfate columns were rinsed twice using 10 mL dichloromethane between each repetition. Solvents were evaporated and 2 mL acetonitrile was added for HPLC or GC analysis. Samples were analyzed for spinetoram residues using a 2690 separator module HPLC, with a 2487 dual-wavelength absorbance detector (Waters, Milford, MA, USA). A C18 reserved-phase column with 4.6 mm bore and 5 mm particle size was used. Flow rate was set at 0.3 mL/minute. The mobile phase was started at 90:10 water:acetonitrile with formic acid (0.1%) and reduced to 70:30 between 12 and 13 min at 20 °C. The detector was set to monitor 745.86 m/z for spinetoram. Acetamiprid and phosmet were analyzed using GC/MSD (Agilent 6890 Gas Chromatograph with a 5973 N Mass Spectra Detector (MSD); Agilent Technologies, Santa Clara, CA) that was equipped with a Zebron ZB-5ms 30 m, 0.25 mm I.D., and a 0.25 µm film thickness. For the GC/MSD analysis settings, the oven was held at 115 °C for five minutes with an increase of 9 °C per minute to 280 °C, followed by an increase of 30 °C per minute to 310 °C. The MSD transfer line was held at 285 °C. The mass spectrometer was set to monitor for ions according to Table 2. The injector was rinsed three times with acetone and three times with dichloromethane between and also before each injection. All compounds were quantified against a standard curve, and recovery data recorded as μg of AI per gram (ppm) of plant substrate.

### 2.4. Statistical Analysis

#### 2.4.1. Bioassay

Mean adult mortality was compared using a repeated-measures ANOVA on square root arcsine-transformed data ((arcsin(x))1/2) to meet normality and homogeneity assumptions. Analysis was performed using PROC MIXED, with the Kenward–Rogers degree of freedom calculation method. Data from 5 days of exposure were excluded from this analysis due to the inability to meet these assumptions. The factor exposure time (1 or 3 day) was treated as the repeated measure and each plastic container served as an experimental unit and was subjected to this repeated measures class. The factor run was treated as a random factor. The two error terms for the model were an interaction between run, insecticide, and rainfall amount, and an interaction between run, insecticide, rainfall amount, and observation day. Post-hoc Tukey’s honestly significant difference (HSD) test was used to assess pairwise comparisons between adult mortality at different rainfall intensities for an insecticide treatment, between different exposure days of an insecticide and rainfall treatment, and differences between adult mortality for an insecticide, rainfall amount, and observation treatment combination with the appropriate untreated control. All tests were done using α = 0.05. All eggs, larvae, and pupae that were found between insecticide and simulated precipitation rate treatment combinations were analyzed using ANOVA. Toxicity was determined by separately comparing total numbers of small larvae, large larvae, pupae, and total of individuals recovered between each insecticide and rainfall treatment combinations each year using a two-way ANOVA in PROC MIXED. The two error terms for the 2018 model were an interaction between run, insecticide, and rainfall amount, and an interaction between run, insecticide, rainfall amount, and observation day. Data were tested for normality and homogeneity assumptions using Shapiro–Wilk and Levene’s test, respectively. Square root transformations of data were done if necessary to meet assumption requirements. Datasets that did not meet homogeneity assumptions were then run with the REPEATED command in PROC MIXED, testing every main effect and their interactions. A model was then chosen based on the lowest Akaike information criterion (AIC) value. Kenward–Rogers degree of freedom calculation test was used to correct for the possibility of artificial inflations. A ranked test was used on data that could not be normalized. Required transformations used in each stage are listed in the results. Means separation between insecticide treatment and application time combinations were done using Tukey’s HSD test. Each run was treated as a random factor in all analyses. All tests were run with α = 0.05 and conducted using SAS software 9.4.

#### 2.4.2. Residue

Data were checked for normality and homogeneity using Shapiro–Wilk and Levene’s test. Appropriate transformation was done to data to meet normality and homogeneity assumptions. Transformations are listed with residue results. Rainfall effects on fruit surface and subsurface residues were analyzed using a one-way ANOVA separately between rainfalls for each insecticide treatment and plant organ. Multi-comparison was done using a Dunnett’s test to determine differences between rainfall treatments and the control (0 mm rainfall). All tests were evaluated at α = 0.05 and performed using SAS software 9.4.

## 3. Results

### 3.1. Adult Mortality

**Figure 1:**
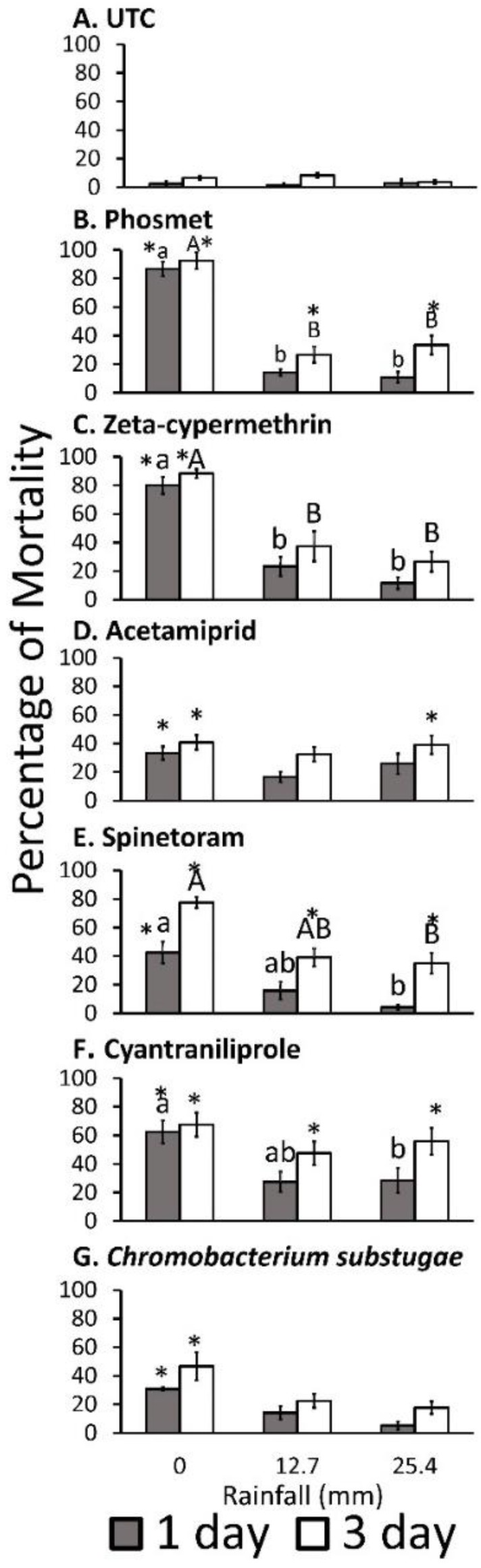
Mean ± SEM percent adult mortality for each insecticide and rainfall treatment combination on each observation day, 2018. Data were analyzed using repeated measures three-way ANOVA. Different lowercase letters indicate significant differences on 1 day observation, while capital letters indicate significant differences on 3 day observations. Asterisks show significant differences between insecticide with observation day and rainfall treatment combination and the untreated with the appropriate observation day and rainfall treatment combination using a Tukey’s honestly significant difference (HSD) post-hoc test (*). All tests were evaluated at α = 0.05.

Insecticide, rainfall amount, and observation day affected adult mortality. The repeated measures ANOVA indicated significant effects of insecticide, rainfall amount, observation day main effects, and insecticide × rainfall amount, insecticide × observation day, rainfall amount × exposure day, and insecticide × rainfall amount × observation on the percentage of adult mortality (Table 3). The several pairwise comparisons showed differences in adult mortality between rainfall and insecticide, between observation day and insecticide, and between observation day and rainfall treatment combinations, and among insecticide × rainfall amount × observation day combinations with the parallel untreated controls. Adult mortality at all insecticide and observation days of 0 mm rainfall was significantly higher than the untreated control (Figure 1). Phosmet and cyantraniliprole showed significantly higher adult mortality after 12.7 and 25.4 mm rainfall only for 3 days of exposure compared with the untreated control. Acetamiprid showed significantly higher mortality only for 25.4 mm rainfall after 3 days of exposure, whereas zeta-cypermethrin and *C. subtsugae* did not demonstrate significant differences in adult mortality on any observation days after 12.7 or 25.4 mm rainfall compared with the untreated control. Adult mortality of flies treated with phosmet and zeta-cypermethrin treated with 12.7 and 25.4 mm rainfall were significantly lower when compared with 0 mm at all periods of exposure days. Adult mortality of flies treated with spinetoram and cyantraniliprole was only significantly lower, with 25.4 mm rainfall compared with 0 mm. Flies treated with phosmet and cyantraniliprole demonstrated significantly higher adult mortality at 12.7 and 25.4 mm rainfall after 3 days of exposure compared with 1 day of exposure (Figure 1B,F), whereas spinetoram caused significantly higher adult mortality at all rainfall treatments after 3 days of exposure (Figure 1E).

### 3.2. Immature Stage Survival

Insecticides, rainfall amount, and their interaction affected the number of all SWD immature life stages (Table 4). The total numbers of SWD immature life stages depended on interactions between insecticide and rainfall amount. The numbers of small larvae recovered from samples were affected by rainfall only on samples treated by phosmet and *C. subtsugae,* while rainfall did not affect small larvae numbers on samples treated with zeta-cypermethrin, acetamiprid, spinetoram, and cyantraniliprole. Small larvae collected from samples treated with spinetoram and 25.4 mm of simulated rainfall were significantly lower than those from the untreated control.

Numbers of large larvae recovered from samples treated with phosmet, zeta-cypermethrin, spinetoram, and *C. subtsugae* significantly increased with rainfall amount. At 0 mm of simulated rainfall, all samples treated with insecticides were significantly lower than the untreated control by 54–92%. The lowest numbers of large larvae were found in samples treated with phosmet, followed by zeta-cypermethrin, cyantraniliprole, spinetoram, acetamiprid, and *C. subtsugae.* Large larvae collected from acetamiprid-treated shoots were not significantly lower compared with zeta-cypermethrin and spinetoram. At 12.7 mm of simulated rainfall, all insecticide treatments but *C. subtsugae* were significantly lower than the untreated control, although reductions were only 40–58% lower. Phosmet demonstrated the lowest level of large larvae compared with other insecticide treatments, followed by spinetoram, cyantraniliprole, acetamiprid, and zeta-cypermethrin. Large larvae numbers were significantly lower for all insecticides but *C. subtsugae* treated at 25.4 mm of simulated rainfall. Large larvae numbers decreased 45–68% compared with the untreated control. Acetamiprid demonstrated the lowest large larvae number counts, although not significantly different among all insecticides treated with 25.4 mm of simulated rainfall.

Phosmet was the only treatment where the number of pupae were significantly affected by simulated rainfall. Pupae counts among insecticide treatments were not significantly lower compared with the untreated control at 0 and 12.7 mm of simulated rainfall. At 25.4 mm of simulated rainfall, only acetamiprid and spinetoram had significantly lower number of pupae compared with the untreated control.

Simulated rainfall significantly affected samples treated with phosmet, zeta-cypermethrin, and *C. subtsugae* total numbers of SWD immature life stage counts. Total survival numbers increased as these samples were treated with 12.7 mm of simulated rainfall. At 0 mm of simulated rainfall, all insecticide treatments had a significantly lower total number of individuals than the untreated control. At both 12.7 and 25.4 mm of simulated rainfall, all insecticide treatments but *C. subtsugae* resulted in significantly lower counts compared with the untreated control. At 12.7 mm of simulated rainfall, spinetoram demonstrated the lowest total counts, whereas at 25.4 mm of simulated rainfall, acetamiprid demonstrated the lowest total counts.

### 3.3. Insecticide Residues Analysis

**Figure 2:**
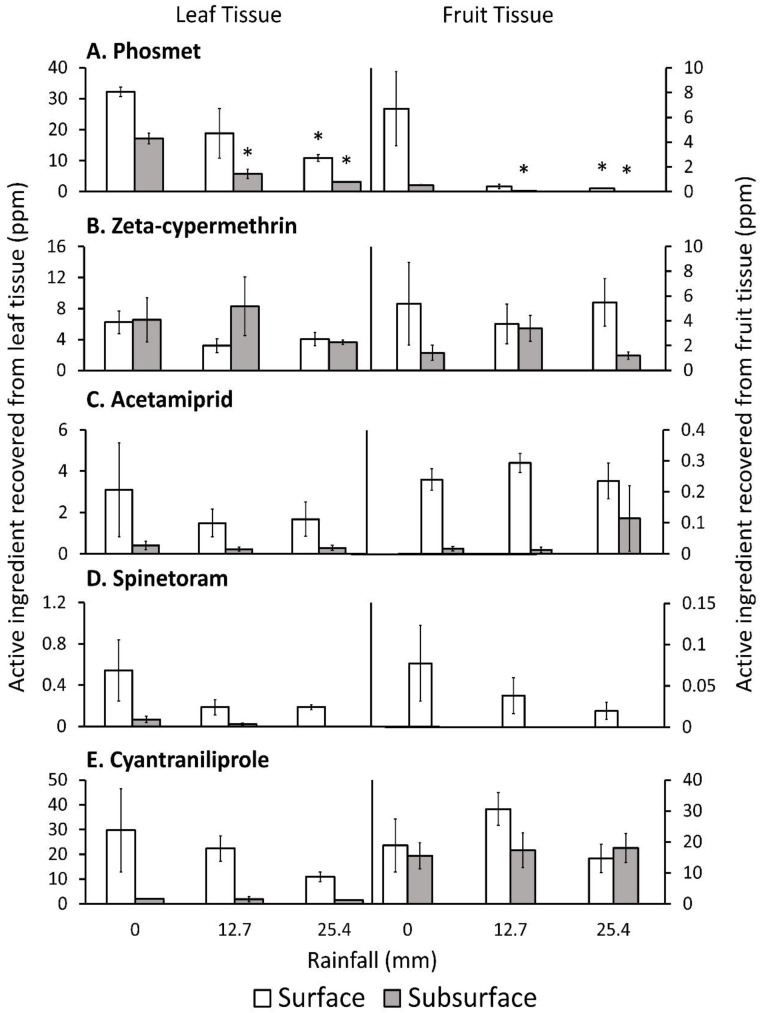
Mean ± SEM of residues collected from tart cherry leaf and fruit surface and subsurface for each rainfall treatment, 2018. Data were analyzed using ANOVA and data that showed significant differences were then tested using a Dunnett’s test. Asterisks (*) show significant differences between residues collected from rainfall treatment and no rainfall treatment (0 mm) from the same insecticide treatment and plant tissue at α = 0.05.

Phosmet amounts recovered from the leaf surface were not significantly different after 25.4 mm of simulated rainfall compared with 0 mm of rainfall (Figure 2, Table 5). However, post-hoc tests indicated leaf surface residues recovered from 25.4 mm of rainfall was significantly lower than the untreated control (0 mm) by ~66%. Residues recovered from the leaf subsurface at all rainfall amounts were significantly lower than 0 mm by >66%. Phosmet residues collected from the fruit surface were significantly lower after 25.4 mm of rainfall compared with 0 mm by 96%. However, phosmet residues collected from the fruit surface after 12.7 mm of rainfall were lower by 93%, but the post-hoc test showed these differences were not significant (t = −2.79 df_denum_ = 6; *p* = 0.0548). Phosmet residues collected from the fruit subsurface were significantly lower for all rainfall treatments compared with 0 mm by >88%.

Zeta-cypermethrin, acetamiprid, cyantraniliprole, and spinetoram residues recovered from rainfall treatments were not significantly different compared with 0 mm of rainfall for all plant parts. However, cyantraniliprole residues recovered from leaf surface showed a numerical decrease after simulated rainfalls; whereas, spinetoram residues collected from the leaf surface, leaf subsurface, and fruit surface showed a numerical decrease after being treated with rainfall.

## 4. Discussion

This study demonstrated the variable effects of rain on the residues of insecticides on tart cherry fruit and leaves, and their ability to cause SWD adult mortality and immature development. Some of the most commonly used insecticides, such as phosmet, zeta-cypermethrin, and spinetoram demonstrated that their adulticide activity is highly sensitive to the effects of rainfall.

Acetamiprid was the most resistant to wash-off from simulated rainfall based on the residue results and low SWD immature stage survival. Acetamiprid’s low adulticide action was consistent with previous results against SWD [9]. In addition, low neonicotinoid adulticide results were consistent with other studies done on codling moth (*Cydia pomonella*) and Japanese beetles (*Popillia japonica*); however, the neonicotinoids demonstrated other forms of control than just mortality, such as feeding or oviposition deterrence [17,19,22]. Acetamiprid log P (log Kow) is the lowest of all compounds tested for residues (0.8), suggesting high penetrative potential in plant tissues. Acetamiprid’s residues in fruit and leaf sub-surfaces likely serves as a protected refuge from the negative effects of rainfall. These fruit subsurface residues also provide toxic exposure to SWD larvae and eggs, likely explaining the noticeable SWD control even after high amounts of simulated rainfall. In addition, previous studies have shown acetamiprid to possess curative action against SWD in tart cherries [23]. Thus, it may be beneficial to consider including acetamiprid in spray programs during periods with high risk of rainfall when controlling this pest.

Simulated rainfall adversely affected SWD control using zeta-cypermethrin and *C. subtsugae.* Zeta-cypermethrin adulticide levels were different from previous studies, which demonstrated high inherent toxicity even after rainfall [16,17]. Residue results indicated that active ingredients were less sensitive to wash-off by simulated rainfall; however, spatial distribution of the compound on plant tissues may have been affected, thus decreasing toxicity to the target pest. Although adult mortality from *C. subtsugae* samples were not affected by simulated rainfall, immature survival was adversely affected. Unfortunately, residue samples for *C. subtsugae* could not be analyzed, thus it is not clear whether these control decreases were caused by heavy reduction of active ingredient, low inherent toxicity, or low larvicidal activity.

Although phosmet control and residues were sensitive to wash-off from rainfall, phosmet still demonstrated better efficacy against SWD compared with the untreated control. This high inherent toxicity was consistent with previous studies on Japanese beetles and codling moth, which demonstrated leaf defoliation and live larvae to be lower than the untreated control even after simulated rainfall [19,22]. It is noteworthy that phosmet is most effective in acidic water (pH = 5) and is hydrolyzed by basic pH levels, causing its effectiveness to be compromised and have shorter half-time periods; thus, this process may help explain why phosmet’s effectiveness was compromised and lower residues were recovered [24]. Azinphos-methyl, another organophosphate, had half-time periods, that were less sensitive to neutral pH (7) than phosmet and were the same at pH = 9 [24]. Thus, active ingredient behavior to rainfall within this insecticide class should not be generalized.

Simulated rainfall moderately affected cyantraniliprole. Adulticide action was affected by simulated rainfall; its effects on immature stages and the residue results were less affected by rainfall. Adulticide results were consistent with previous studies, which demonstrated that rainfall adversely affected cyantraniliprole SWD adulticide action on blueberries [16]. However, another study demonstrated codling moth control using cyantraniliprole to be less sensitive to simulated rainfall [19]. Based on its residue profiles, cyantraniliprole may possess other control activity besides adulticide action, such as oviposition deterrence, ovicidal, or larvicidal activity. To minimize the effect of rainfall, other studies have suggested that adding an adjuvant may help increase cyantraniliprole adulticide action when rainfall occurs [16].

Simulated rainfall adversely affected spinetoram’s adulticide action and residues, but its lethality to SWD immature stages was less affected. Although spinetoram residues from our 2018 studies were not affected by rainfall, there was a noticeable decline numerically. Spinetoram’s inherent toxicity against SWD was consistent with a previous study with codling moth, causing lower larvae survival and SWD adulticide action even after simulated rainfall [16,19]. Spinetoram’s inherent toxicity may have to do with its high toxicity based on its LC_50_ and LC_90_ compared with other compounds [25]. These results imply that beside adulticide activity, spinetoram possessed ovicidal and larvicidal activity even at lower concentrations. It is noteworthy that adult mortality increased over time after spinetoram, phosmet, and cyantraniliprole samples were treated with simulated rainfall. Thus, continuous exposure to lower residues may still provide control against SWD.

Precipitation effects on chemical crop protection are complex and not yet fully understood. To date, most studies associated with insecticides and SWD control have focused on the effects of precipitation on adult mortality without directly measuring residue losses from the plant or considering impacts on immature life stages [16]. This study provides immature survival and residue results as well as adult mortality; thus, it helps provide additional information on other modes of activity that may occur after precipitation events. It is noteworthy that precipitation effects on crop protection systems may extend beyond the direct effects on pesticide residue and the target pest. A recent study demonstrated rainfall-affected tree volatiles associated with repellency and attraction of codling moth in apples, possibly causing complicated interchanges which can affect crop preferences in herbivores [26]. Due to the dynamics among plants, insects, chemicals, precipitation, and interactions among these systems, it is possible that precipitation’s effects on crop protection may extend to more than simply washing off insecticides from crops. This study provides information on how different insecticide compounds at labelled field rates behave after various rainfall amounts. It also provides additional emphasis that other modes of activities besides mortality may contribute to SWD management programs following precipitation events. Thus, tart cherry growers may use these results as a basis for informed decisions on insecticide application and reapplication before and after rainfall events.

## 5. Conclusions

This study demonstrates the differences of rainfall effects on residue wash-off, and adult and immature SWD toxicity among several insecticide active ingredients. Rainfall significantly affected major active ingredients used against SWD, such as phosmet, zeta-cypermethrin, and spinetoram; acetamiprid and cyantraniliprole, active ingredients recognized for being less adulticidal against SWD, were less sensitive to rainfall. Our research demonstrates that active ingredients behave differently under rainfall and these differences may be valuable to be considered in SWD spraying programs.

## Figures and Tables

**Table 1:** Active ingredient, insecticide group, formulation brand, manufacture, rate, and field rate of formulation used.

Active Ingredient	Insecticide Group	Trade Name	Manufacture	Rate	Field Rate
phosmet	organophosphate	Imidan 70W	Gowan Corporation, Yuma, AZ	1680 g AI ha^−1^	2.125 lb. acre^−1^
*Chromobacterium subtsugae*	biopesticide	Grandevo DF	Marrone Bio Innovations, Inc., Davis, CA	1008 g AI ha^−1^	3 lb. acre^−1^
cyantraniliprole	anthranilic diamide	Exirel 10SE	DuPont, Wilmington, DE	100.6 mL AI ha^−1^	13.5 fl. oz. acre^−1^
acetamiprid	neonicotinoid	Assail 30SG	United Phosphorous Inc., Abingdon, VA	111.3 g AI ha^−1^	5.3 oz. acre^−1^
zeta-cypermethrin	pyrethroid	Mustang Maxx .8EC	FMC Corp., Philadelphia, PA	28 g AI ha^−1^	4 fl. oz. acre^−1^
spinetoram	spinosyn	Delegate 25WG	Dow AgroSciences LLC, Indianapolis, IN	105 g AI ha^−1^	6 oz. acre^−1^
* 2-hydroxy-1,2,3-propanetricarboxylic acid	adjuvant	Tri-fol	Wilbur–Ellis Company LLC, Fresno, CA	0.62–2.5 mL L^−1^	0.5–2 pint per 100 gal

* was only added to the phosmet treatment.

**Table 2:** Ions monitored in a mass spectrometer and the limit of detection (LOD) and limit of quantitation (LOQ) for each treatment compound in 2017 and 2018 residue analyses.

Compound	M+H (m/z)	Qualifier (m/z)	LOD (μg/g)	LOQ (μg/g)
phosmet	161	160	0.015	0.05
zeta-cypermethrin	209	163	0.005	0.010
acetamiprid	223	152	0.015	0.05
spinetoram	784.5	142.4	0.121	0.40
cyantraniliprole	475	286	0.005	0.010

**Table 3:** Statistical variables of fixed effects and nested interactions for repeated measures analyses of adult mortality observed 1, 3, and 5 days after various insecticides were treated with 0, 12.7, and 25.4 mm of simulated rainfall in the 2018 trial.

Effect	Numerator df	Denumerator df	F-Value	Pr > F
Insecticide	6	185	25.4	<0.0001
Rainfall	2	185	67.13	<0.0001
Insecticide × Rainfall	12	185	5.43	<0.0001
Observation Day	1	186	251.99	<0.0001
Insecticide × Observation Day	6	186	6.91	<0.0001
Rainfall × Observation Day	2	186	6.67	0.0016
Insecticide × Rainfall × Observation Day	12	186	1.85	0.0438

**Table 4:** Mean ± SE number of *Drosophila suzukii* small larvae (<2mm), large larvae (>2mm), pupae, total individuals recovered from samples after 9 days since infestation with 6 females and 6 males, 2018. Shoots were treated with simulated rainfall (0, 12.7, and 25.4 mm). Means separation was done using Tukey’s HSD test. Different lowercase letters indicate significant differences within a column, whereas different capital letters indicate significant differences within a row at a developmental stage. All tests were done at α = 0.05. Data shown are untransformed values.

Treatment	Small Larvae			Large Larvae			Pupae			Total		
	0	12.7	25.4	0	12.7	25.4	0	12.7	25.4	0	12.7	25.4
untreated control	1.80 ± 0.76 ^aA^	2.30 ± 0.99 ^aA^	2.33 ± 0.80 ^aA^	37.90 ±3.71 ^aA^	37.50 ± 2.20 ^aA^	44.78 ± 6.46 ^aA^	4.30 ± 1.04 ^aA^	5.80 ± 1.34 ^aA^	6.78 ± 1.62 ^aA^	44.00 ± 4.34 ^aA^	45.60 ± 2.70 ^aA^	53.89 ± 6.67 ^aA^
phosmet	0.00 ± 0.00 ^bB^	0.30 ± 0.17 ^aAB^	1.00 ± 0.60 ^abA^	2.80 ± 0.76 ^dB^	15.70 ± 4.07 ^bA^	18.00 ± 3.82 ^bcA^	2.00 ± 0.86 ^aB^	6.30 ± 1.57 ^aA^	6.00 ± 1.45 ^abA^	4.80 ± 1.62 ^cB^	29.40 ± 4.85 ^bA^	25.00 ± 3.75 ^bcA^
zeta-cypermethrin	0.40 ± 0.27 ^abA^	0.22 ± 0.21 ^aA^	0.60 ± 0.22 ^abA^	7.30 ±1.73 ^cdB^	22.22 ± 2.87 ^bA^	24.50 ± 4.90 ^abcA^	3.00 ± 1.09 ^aA^	3.56 ± 0.98 ^aA^	4.00 ± 0.86 ^abA^	10.70 ± 2.34 ^bcB^	26.00 ± 2.82 ^bA^	29.00 ± 4.86 ^bcA^
acetamiprid	1.10 ± 0.46 ^aA^	2.10 ± 0.94 ^aA^	1.20 ± 0.33 ^aA^	10.90 ± 2.65 ^bcA^	19.10 ± 2.06 ^bA^	13.90 ± 3.76 ^cA^	3.80 ± 1.28 ^aA^	2.80 ± 0.49 ^aA^	2.40 ± 0.68 ^bA^	15.80 ± 3.83 ^bA^	24.00 ± 2.82 ^bA^	17.50 ± 3.99 ^cA^
spinetoram	0.20 ± 0.13 ^abA^	0.30 ± 0.21 ^aA^	0.00 ± 0.00 ^bA^	10.60 ± 1.20 ^bcB^	16.00 ± 1.83 ^bAB^	20.30 ± 2.77 ^bcA^	3.40 ± 1.09 ^aA^	4.70 ± 1.41 ^aA^	2.10 ± 0.72 ^bA^	14.20 ± 2.49 ^bA^	21.00 ± 1.43 ^bA^	22.40 ± 2.38 ^bcA^
cyantraniliprole	0.50 ± 0.40 ^abA^	0.60 ± 0.31 ^aA^	0.80 ± 0.33 ^abA^	9.80 ± 2.77 ^bcA^	16.90 ± 3.12 ^bA^	15.90 ± 3.32 ^bcA^	2.10 ± 0.48 ^aA^	3.60 ± 1.18 ^aA^	3.10 ± 0.81 ^abA^	12.40 ± 2.70 ^bcA^	21.10 ± 2.90 ^bA^	19.80 ± 3.71 ^cA^
*Chromobacterium subtsugae*	0.10 ± 1.07 ^abB^	2.00 ± 0.63 ^aA^	2.63 ± 1.38 ^aA^	17.40 ± 5.09 ^bB^	26.70 ± 3.28 ^aAB^	32.00 ± 3.65 ^abA^	3.60 ± 1.28 ^aA^	5.70 ± 1.86 ^aA^	4.63 ± 1.39 ^abA^	21.10 ± 2.45 ^bB^	34.40 ± 4.94 ^abAB^	39.25 ± 4.47 ^abA^
	Rank			sqrt(x), unequal variance by rain			sqrt(x), unequal variance by ins*rain			sqrt(x), unequal variance by rain		
insecticide (F; df; P)	8.69; 6, 184; <0.0001			27.11; 6, 156; <0.0001			2.75; 6, 66.5; 0.0191			24.75; 6, 173; <0.0001		
rainfall (F; df; P)	5.47; 2, 184; 0.0049			29.23; 2, 118; <0.0001			3.55; 2, 102; 0.0324			29.43; 2, 119; <0.0001		
insecticide × rainfall (F; df; P)	1.61; 12, 184; 0.0927			1.66; 12, 150; 0.0809			0.99; 12, 57.5; 0.4719			2.04; 12, 152; 0.0244		

**Table 5:** Statistical variables of ANOVA fixed effects for residues recovered from leaf surface, leaf subsurface, fruit surface, and fruit subsurface after 0, 12.7, and 25.4 mm of simulated rainfall from 2018 trial.

Treatment		Leaf Tissue		Fruit Tissue	
		Leaf Surface	Leaf Subsurface	Fruit Surface	Fruit Subsurface
phosmet	(F; df; P)	5.11; 2, 6; 0.0506	32.35; 2, 6; 0.0006	5.61; 2, 6; 0.0423	102.53; 2, 6; <0.0001
	transformation	- ^a^	-	sqrt(x − 0.1)	-
zeta-cypermethrin	(F; df; P)	1.93; 2, 6; 0.2253	0.73; 2, 6; 0.5192	0.17; 2, 6; 0.8507	2.80; 2, 6; 0.1387
	transformation	-	-	-	-
acetamiprid	(F; df; P)	0.97; 2, 6; 0.7062	0.38; 2, 6; 0.38	0.58; 2, 6; 0.5874	0.82; 2, 6; 0.4857
	transformation	-	-	-	sqrt(x + 0.1)
spinetoram	(F; df; P)	1.36; 2, 6; 0.3264	3.60; 2, 6; 0.0941	0.97; 2, 6; 0.4313	ND ^b^
	transformation	-	-	-	-
cyantraniliprole	(F; df; P)	0.86; 2, 6; 0.4697	0.09; 2, 6; 0.9114	1.70; 2, 6; 0.2608	0.07; 2, 6; 0.9351
	transformation	-	-	-	-

^a^ No transformation. ^b^ Not detected.
